# Wheat and barley dehydrins under cold, drought, and salinity – what can LEA-II proteins tell us about plant stress response?

**DOI:** 10.3389/fpls.2014.00343

**Published:** 2014-07-09

**Authors:** Klára Kosová, Pavel Vítámvás, Ilja T. Prášil

**Affiliations:** Plant Stress Biology and Biotechnology, Department of Plant Genetics, Breeding and Product Quality, Crop Research InstitutePrague, Czech Republic

**Keywords:** dehydrin dynamics, proteins, transcripts, abiotic stress, barley, wheat

## Abstract

Dehydrins as a group of late embryogenesis abundant II proteins represent important dehydration-inducible proteins whose accumulation is induced by developmental processes (embryo maturation) as well as by several abiotic stress factors (low temperatures, drought, salinity). In the review, an overview of studies aimed at investigation of dehydrin accumulation patterns at transcript and protein levels as well as their possible functions in common wheat (*Triticum aestivum*), durum wheat (*T. durum*), and barley (*Hordeum vulgare*) plants exposed to various abiotic stress factors (cold, frost, drought, salinity) is provided. Possible roles of dehydrin proteins in an acquisition and maintenance of an enhanced frost tolerance are analyzed in the context of plant developmental processes (vernalization). Quantitative and qualitative differences as well as post-translational modifications in accumulated dehydrin proteins between barley cultivars revealing differential tolerance to drought and salinity are also discussed. Current knowledge on dehydrin role in wheat and barley response to major dehydrative stresses is summarized and the major challenges in dehydrin research are outlined.

## INTRODUCTION

Abiotic stress factors – cold, frost, drought, salinity – severely limit plant growth and development as well as the final yield in crops including cereals from the tribe Triticeae. Low temperatures, drought, and salinity represent stress factors associated with plant cell dehydration. Dehydration stress factors induce profound cellular response aimed at an elimination of water loss. Plant cell response to dehydration includes an accumulation of osmotically active compounds including hydrophilic proteins such as dehydrins. Several physiological studies focused on plant stress response have reported a positive relationship between the level of accumulation of dehydrin transcripts or proteins and plant stress tolerance (Kosová et al., 2010). More tolerant cultivars or genotypes usually reveal a higher level of dehydrin transcripts or proteins than the less tolerant ones although the relationship between plant stress tolerance and dehydrin transcript or protein accumulation is not always obvious (linear) due to a complex nature of plant stress tolerance mechanisms.

The aim of the study lies in a summary of the research focused on dehydrin transcript and protein accumulation in wheat and barley plants exposed to various abiotic stresses with a dehydrative component – cold, drought, salinity. We have tried to summarize recent results gained on cultivated common wheat (*Triticum aestivum*), durum wheat (*T. durum*) and barley (*Hordeum vulgare*) exposed to cold, frost, drought, and salt stress factors.

## DEHYDRINS IN WHEAT AND BARLEY

There has already been published a complete annotation of barley genome sequence (The International Barley Genome Sequencing Consortium, 2012) while common wheat (*T. aestivum*) complete genome sequencing is still in progress (). Barley (*H. vulgare*) represents the relatively most tolerant cultivated Triticeae species with respect to dehydrative stresses, especially drought and salinity (Colmer et al., 2006). There have been 13 dehydrin genes *Dhn1* to *Dhn13* identified in the barley genome (Tommasini et al., 2008). Common wheat genome is much larger than in barley due to its allohexaploid nature resulting in the presence of orthologs and also paralogs (Sarhan et al., 1997; Danyluk et al., 1998). Recently, Wang et al. (2014) have identified 54 dehydrin unigenes in common wheat genome by a search of wheat EST database.

Dehydrin structural types and expression patterns: both barley and wheat contain four (K_n_, SK_n_, Y_n_SK_m_, K_n_S) out of five dehydrin structural types (Close, 1997) while they lack dehydrins of Y_n_K_m_ type (**Table 1**). The largest group of dehydrins in barley (10 out of 13 *Dhn* genes) as well as in common wheat belong to Y_n_SK_m_ type which encompass basic dehydrins induced by strong dehydration stresses (drought, salt, frost) as well as by abscisic acid (ABA) due to an occurrence of several ABRE elements in their promoters (Choi et al., 1999). Some of Y_n_SK_m_ type dehydrins are embryo-specific such as barley *Dhn12* (Choi and Close, 2000). K_n_ type dehydrins DHN5 in barley and several K_n_ type dehydrins (WCS120 family as well as low-molecular K_n_ type dehydrins such as WDHN13) in common wheat are induced by both cold and drought as well as by ABA (Choi et al., 1999; Tommasini et al., 2008; Wang et al., 2014). There has also been described an induction of barley *Dhn5* by moderate levels of salicylic acid (SA) up to 0.25 mM while an inhibition by SA levels higher than 0.4 mM (Sun et al., 2009). For K_n_ type dehydrins with multiple copies of K-segment such as barley DHN5 (K_9_) and wheat WCS120 (K_6_), a cryoprotective activity has been reported (Houde et al., 1995; Bravo et al., 2003) similarly to Y_n_K_m_ type dehydrins such as peach PCA60 (Wisniewski et al., 1999). Acidic SK_3_ dehydrins include DHN8 in barley and WCOR410 family (WCOR410a,b,c) in common wheat and are induced predominantly by cold (Danyluk et al., 1994, 1998; Choi et al., 1999). Wheat SK_n_ type dehydrins can be induced by dehydration, ABA, methyl jasmonate (MeJA), gibberellins (GA), and SA (Zhu et al., 2014). KS type dehydrins include DHN13 in barley and at least one ortholog in wheat. KS type dehydrins seem to represent a specific group of small dehydrin proteins induced by chilling exclusively in reproductive tissues such as anthers (Rodriguez et al., 2005; Wang et al., 2014). Phylogenetic analysis of protein sequences of 13 *Dhn* genes in barley has revealed a high sequence similarity between the 10 dehydrin proteins of Y_n_SK_m_ type (DHN1,2,3,4,6,7,9,10,11,12) while the remaining three dehydrin proteins DHN5 (K_9_), DHN8 (SK_3_), and DHN13 (KS) revealed significant differences with respect to Y_n_SK_m_ type dehydrins as well as each other (Karami et al., 2013). DHN13 seems to be most distant with respect to the other barley dehydrin proteins both at protein sequence and expression levels (Karami et al., 2013).

**Table 1:** An overview of dehydrins in barley (*Hordeum vulgare*), common wheat (*Triticum aestivum*), and durum wheat (*Triticum durum*) listed according to their structural types described in Close (1997) including their expression patterns as well as characteristic features.

Structural type	Expression pattern	Characteristic features	Corresponding dehydrins in barley and wheat
K_n_	Low temperatures (cold, frost), drought, salt, ABA, SA	Cryoprotective	Barley: DHN5 (K_9_)
			Common wheat: WCS120 family – WCS120 (K_6_); low-molecular K_n_ types – WDHN13 (K_2_)
SK_n_	Cold (chilling, frost)	Acidic; membrane lipid interactions	Barley: DHN8 (SK_3_)
			Common wheat: WCOR410a,b,c (SK_3_)
K_n_S	Chilling-induced	Reproductive tissues (anthers)	Barley: DHN13 (KS)
			Common wheat: at least one ortholog to barley DHN13 (KS type)
Y_n_SK_m_	Strong dehydrative stresses (drought, frost, salt), ABA, GA, MeJA, SA	Basic; accumulation in cytoplasm and nucleus; phosphorylation of S-segment (NLS); allelic variation (barley *Dhn4*); some are embryo-specific	Barley: DHN1,2,3,4,6,7,9,10,11,12 Common wheat: several drought- and ABA-inducible homologs, e.g., RAB15 (YSK_2_) Durum wheat: DHN5 (YSK_2_)
Y_n_K_m_	Cold	Antifreeze, cryoprotective	Not present in barley and wheat genomes

*ABA, abscisic acid; GA, gibberellic acid; MeJA, methyl jasmonate; NLS, nuclear localization signal; SA, salicylic acid.*

Dehydrins are intracellular proteins which accumulate to high levels in cell cytoplasm but they can also be found in several organelles. Dehydrins containing a stretch of serine residues, the S-segment, can be regulated via phosphorylation of the S-segment. Phosphorylated S-segment has been reported to function as a nuclear localization signal (NLS) for DHN5 (YSK_2_) in durum wheat (Brini et al., 2007a). However, nuclear localization has also been reported for dehydrin proteins lacking the S-segment such as WCS120 in cold-treated wheat crown tissue (Houde et al., 1995). At tissue and organ level, comparison of vegetative organs (leaves, crowns, roots) revealed the relatively highest dehydrin protein accumulation in crowns followed by leaves and relatively lowest accumulation in roots when expressed per unit amount of organ fresh weight in salt-treated barley plants (Kosová et al., 2013a). Similarly, Houde et al. (1995) have also found the relatively highest accumulation of WCS120 proteins in wheat crown tissue upon cold since crowns represent the crucial organ for the whole plant survival. At tissue level, immunoanalyses have revealed an enhanced accumulation of dehydrins in xylem cells associated with vessel elements both in wheat (Houde et al., 1995; Danyluk et al., 1998) and barley (Bravo et al., 1999).

## DEHYDRIN PROTEIN FUNCTION UPON ABIOTIC STRESS

### BIOCHEMICAL STUDIES (*IN VITRO* STUDIES)

There have been reported several protective functions for isolated wheat and barley dehydrin proteins in *in vitro* assays. Comparative studies using wild-type and mutant forms of dehydrin proteins lacking one of the conserved dehydrin sequences, i.e., Y-, S-, K- or Φ- segments, have revealed a crucial role of the amphipathic lysine-rich K-segment in dehydrin chaperone (heat stress) and cryoprotective functions as reported for K_n_ type dehydrins with multiple copies of K-segment such as WCS120 (K_6_) in common wheat (Houde et al., 1995) and DHN5 (K_9_) in barley (Bravo et al., 2003), but also for durum wheat DHN-5, a YSK_2_ protein of 227 amino acids (Brini et al., 2010). The protective effect of dehydrin proteins seems to depend on the number of copies of K-segment, as shown in an *in vitro* study on wild-type and mutated forms of durum wheat DHN5 with respect to their protective effects on lactate dehydrogenase (LDH) and β-glucosidase activities under dehydration stress (Drira et al., 2013). The mutated forms of durum wheat DHN5 lacking both copies of K-segment failed in LDH and β-glucosidase protection with respect to DHN5 forms containing at least one K-segment.

### PHYSIOLOGICAL STUDIES (*IN VIVO* STUDIES)

#### Low-temperatures (cold)

Exposure of barley and wheat plants to cold induces two kinds of plant response – cold acclimation, a short-term response aimed at an enhancement of plant frost tolerance, and vernalization, a long-term developmental response aimed at a prevention of a premature transition from a more tolerant vegetative phase to a more sensitive reproductive phase (Kosová et al., 2008a). The major cold-induced dehydrin proteins detected in wheat and barley samples on the immunoblots belong to K_n_ type such as wheat proteins from WCS120 family (Sarhan et al., 1997) and barley DHN5 (Close et al., 1995; Bravo et al., 1999, 2003) although at transcript level, a cold-inducible expression has also been reported for acidic SK_3_ dehydrins in both wheat and barley (Danyluk et al., 1994; Choi et al., 1999; Tommasini et al., 2008). At dehydrin sequence level, there has been found a relationship between allelic variation in *Dhn4* and *Dhn7* gene sequence (a 6 bp insertion in exon 1 of *Dhn4* and a 30 bp deletion in exon 1 of *Dhn7*) and acquired frost tolerance in a set of 30 barley cultivars including both spring and winter growth habits of a wide range of lethal temperature of 50% of the sample (LT50) values (Holková et al., 2010). At transcript and protein levels, there has been repeatedly reported a correlation between dehydrin accumulation and plant acquired frost tolerance in short-term (7 to 21 days) cold acclimation studies (Houde et al., 1992; Vítámvás et al., 2007; Kosová et al., 2008b; Holková et al., 2009). There have been found significant and reproducible differences not only between the contrasting growth habits (spring vs winter), but also at a scale of differentially frost-tolerant winter genotypes when plants were grown under controlled conditions (constant cold temperature and irradiation, neutral photoperiod 12 h day/night). Thus, relative accumulation of selected dehydrin transcripts (*Wcs120*, *Wdhn13* in common wheat) and proteins (WCS120 in common wheat, DHN5 in barley) can be considered a reliable marker of plant frost tolerance under controlled conditions. Under field conditions, the quantitative relationships between dehydrin protein relative accumulation and frost tolerance seem to be more complex and the evaluation of the sum of all dehydrin proteins on the immunoblots rather than a single dehydrin protein seem to provide more reliable results (Prášil et al., 2014). There have also been published several studies dealing with an induction of dehydrin transcript and protein accumulation with respect to a decreasing temperature. Immunoblot analyses have shown that the quantitative differences in dehydrin protein relative accumulation between tolerant and sensitive genotypes found in cold-treated plants can be detected at significantly higher temperatures up to 20^∘^C in highly concentrated protein samples (Vítámvás et al., 2010; Kosová et al., 2013b).

#### Effect of vernalization

Dynamics of WCS120 accumulation under a long-term cold treatment (16 weeks) has been studied in a high frost-tolerant winter wheat Mironovskaya 808 (Vítámvás and Prášil, 2008). Under cold, there have been found high WCS120 protein levels in vernalized plants when exposed to a continuous cold treatment. However, there has been found a differential effect of a deacclimation treatment on dehydrin protein relative accumulation in unvernalized vs vernalized plants. Unvernalized plants are able to reacclimate, i.e., to induce enhanced dehydrin levels upon cold following a deacclimation treatment, while vernalized plants fail to reacclimate. The explanation of the observed difference between unvernalized vs vernalized plants can be based on the results of Seo et al. (2009) in *Arabidopsis thaliana*. In *A. thaliana*, a negative effect of vernalization-induced SOC1 (suppressor of overexpression of constans1) factor on CBF (C-repeat binding factor) transcription factors has been described resulting in an inhibition of CBFs and a downstream *Cor* (*Cold-regulated*) gene expression in vernalized plants. A direct physical interaction of SOC1 protein with CBF promoter regions resulting in an inhibition of CBF gene expression has been found (Seo et al., 2009). In einkorn wheat, there has been described a homolog of *A. thaliana SOC1* gene, *WSOC1*, by Shitsukawa et al. (2007); however, its role remains unclear. There has been described an inhibition effect of the vernalization-induced gene *VRN1* (*Vernalization1*) on the expression of CBFs and downstream *Cor* genes (Dhillon et al., 2010); however, the molecular mechanisms underlying the interaction remain to be elucidated.

#### Effect of sub-zero temperatures (frost)

Frost represents a severe dehydrative stress due to a formation of ice crystals outside the cells resulting in a strong cellular dehydration. Upon a short-term frost treatment (-4^∘^C for 1 h), there has been found an accumulation of high-molecular *Dhn5* as well as several low-molecular *Dhn* transcripts of Y_n_SK_m_ type (*Dhn1, Dhn2, Dhn3, Dhn4, Dhn7, Dhn9*) in barley (Zhu et al., 2000).

#### Drought

Drought induces cellular dehydration and expression of several dehydrins in wheat and barley. In durum wheat, there has been studied an accumulation of YSK_2_ dehydrin DHN5 in two cultivars differing in their drought tolerance (Brini et al., 2007a). There has been found a differential phosphorylation pattern between the two cultivars with the tolerant cultivar revealing a higher extent of phosphorylation than the sensitive one. Phosphorylation is associated with protein differential subcellular localization affecting the final protein function. In wild barley (*H. vulgare* ssp. *spontaneum*) accessions sampled at two sites in Israel and Jordan, there has been found an enhanced expression of several *Dhn* transcripts (*Dhn1,3,5,6,9*) revealing quantitative differences with respect to differential drought tolerance (Suprunova et al., 2004). Similarly, Karami et al. (2013) have reported an induction of several Y_n_SK_m_ dehydrins (*Dhn1, Dhn3, Dhn5, Dhn7,* and *Dhn9*) in barley flag leaf under terminal drought. Relative expression levels of *Dhn3* and *Dhn9* revealed positive correlations with chlorophyll *a* and *b* contents, osmotic adjustment, plant biomass and grain yield, and negative correlations with malondialdehyde and electrolyte leakage levels. In two barley cultivars with a differential tolerance to drought, a Czech spring variety Amulet and Syrian landrace-derived cultivar Tadmor, both quantitative and qualitative differences in low-molecular dehydrin proteins have been found when the plants were subjected to a decreased field water capacity (Škodáček and Prášil, 2011). The qualitative differences in accumulated dehydrin proteins may be caused either by accumulation of different low-molecular *Dhn* genes or by allelic variants of the same gene differing in the copy number of hydrophilic Φ segments and electrophoretic mobility, as described for *Dhn4* (Choi et al., 1999).

#### Salinity

Salinity represents a serious problem in many arid and semi-arid regions. Of cultivated Triticeae, barley (*H. vulgare*) is most tolerant being able to grow up to 250 mM NaCl, common wheat (*T. aestivum*) is less tolerant and durum wheat (*T. durum*) is the least salt-tolerant crop (Colmer et al., 2006). Studies aimed at dehydrin protein accumulation in differentially tolerant genotypes revealed both quantitative and qualitative differences in dehydrin proteins as well as their post-translational modification. Differences in phosphorylation pattern of YSK_2_ dehydrin DHN5 associated with protein nuclear localization have been observed in two differentially salt-tolerant durum wheat cultivars (Brini et al., 2007a). In two barley cultivars with a differential tolerance to high salt levels (300 mM NaCl), both quantitative and qualitative differences in low-molecular dehydrin proteins have been found (Kosová et al., 2013a). The explanation could be analogous to drought, i.e., the qualitative differences may be caused either by accumulation of different low-molecular *Dhn* genes or different allelic variants of the same gene (Choi et al., 1999).

#### Transgenic studies

Several transgenic studies using wheat dehydrins have revealed a positive effect of dehydrin expression on plant stress tolerance. Overexpression of durum wheat DHN5 improved tolerance to osmotic and salinity stresses (200 mM mannitol, 200 mM NaCl) in transgenic *A. thaliana* (Brini et al., 2007b). A pleiotropic effect of DHN5 on expression of several abiotic and biotic stress-responsive genes has been found in transgenic *Arabidopsis* (Brini et al., 2011). Expression of common wheat WCOR410a in strawberry resulted in a 5^∘^C decrease in LT50 values in the transformants with respect to the wild-type (Houde et al., 2004). However, stress tolerance represents a complex trait and the sole overexpression of a single *Lea* gene does not always lead to a significant improvement of stress tolerance as shown by Iturriaga et al. (1992).

## CONCLUSION

Dehydrins have been studied in many plants during the past two decades (Rorat, 2006; Battaglia et al., 2008; Kosová et al., 2010). Cereals from the tribe Triticeae belong to the most studied plants due to their economic importance and adverse effects of abiotic stresses on their productivity. Dehydrins in barley and wheat have already been well characterized at gene and transcript levels (primary sequence, promoter analysis, induction by several factors; Karami et al., 2013; Wang et al., 2014); however, dehydrin proteins, not genes or transcripts, are directly involved in an acquisition of stress tolerance, and, therefore, it is necessary to study dehydrin role in plant stress response at protein level. Dehydrin transcripts respond more rapidly to changes in environment while proteins are more conservative. Comparative studies focused on parallel dynamics of *WCS120* transcript and protein during cold acclimation of common wheat have revealed a much faster induction of *WCS120* transcript upon cold reaching a peak at ca 2 days after an onset of cold treatment while WCS120 protein accumulation reached a peak at ca 21 days after an onset of cold (Ganeshan et al., 2008). Similarly, dehydrin transcript degradation after a temperature increase in a deacclimation treatment is much more rapid than the corresponding protein since dehydrin protein WDHN13 was detected on the immunoblots even at 3 days after the onset of deacclimation while transcript was barely detectable at 1 day of deacclimation (Ohno et al., 2003). Therefore, it is necessary to study the dynamics of dehydrin responses to abiotic stresses at protein level since several post-transcriptional control mechanisms (alternative start codons, natural antisense transcripts) may significantly affect final protein expression. The study at protein level may be complicated by some methodological problems such as a high similarity of barley Y_n_SK_m_ dehydrin protein sequences and thus problems with mass spectrometry identification. Protein post-translational modification such as phosphorylation also significantly affects protein subcellular localization and its final function. There has been found a correlation between dehydrin protein relative accumulation (WCS120, DHN5) and plant maximum acquired frost tolerance in plants subjected to cold (Vítámvás et al., 2007; Kosová et al., 2008b) and even in plants subjected only to mild cold when the plants were grown under controlled temperature (Vítámvás et al., 2010; Kosová et al., 2013b). In field samples, the relationships seem to be more complex and a sum of all detected dehydrin proteins rather than a single protein seems to represent a reliable marker for selection of frost-tolerant materials.

There have already been published several studies on dehydrins in wheat and barley plants. Dehydrins have been well characterized at protein sequence, promoter structure and short-term transcript induction. However, a long-term dynamics of transcripts and proteins with respect to an acquisition of an enhanced stress tolerance as well as dehydrin protein post-translational modifications, cellular localization and their physico-biochemical functions in stress-treated plants remain largely unresolved and represent currently the main challenge to understand dehydrin role in plant stress response.

## AUTHOR CONTRIBUTIONS

Klára Kosová has outlined the idea and prepared the text of a brief summary on current state of dehydrin research in stress-treated wheat and barley plants. Pavel Vítámvás and Ilja T. Prášil contributed to preparation, drafting, critical reading, and publication of the manuscript.

## Conflict of Interest Statement

The authors declare that the research was conducted in the absence of any commercial or financial relationships that could be construed as a potential conflict of interest.
